# DeepSVP: integration of genotype and phenotype for structural variant prioritization using deep learning

**DOI:** 10.1093/bioinformatics/btab859

**Published:** 2021-12-24

**Authors:** Azza Althagafi, Lamia Alsubaie, Nagarajan Kathiresan, Katsuhiko Mineta, Taghrid Aloraini, Fuad Al Mutairi, Majid Alfadhel, Takashi Gojobori, Ahmad Alfares, Robert Hoehndorf

**Affiliations:** Computational Bioscience Research Center (CBRC), Computer, Electrical and Mathematical Sciences & Engineering Division (CEMSE), King Abdullah University of Science and Technology (KAUST), Thuwal 23955-6900, Saudi Arabia; Computer Science Department, College of Computers and Information Technology, Taif University, Taif, Saudi Arabia; Department of Pathology and Laboratory Medicine, King Abdulaziz Medical City (KAMC), Riyadh, Saudi Arabia; Center for Genetics and Inherited Diseases, Taibah University, Almadinah Almunwarah, Saudi Arabia; Supercomputing Core Lab, KAUST, Thuwal, Saudi Arabia; Computational Bioscience Research Center (CBRC), Computer, Electrical and Mathematical Sciences & Engineering Division (CEMSE), King Abdullah University of Science and Technology (KAUST), Thuwal 23955-6900, Saudi Arabia; Department of Pathology and Laboratory Medicine, King Abdulaziz Medical City (KAMC), Riyadh, Saudi Arabia; King Saud bin Abdulaziz University for Health Sciences, King Abdullah International Medical Research Centre, Ministry of National Guard-Health Affairs (MNG-HA), Riyadh, Saudi Arabia; Genetics & Precision Medicine Department, King Abdulaziz Medical City, Ministry of National Guard-Health Affairs (MNG-HA), Riyadh, Saudi Arabia; King Saud bin Abdulaziz University for Health Sciences, King Abdullah International Medical Research Centre, Ministry of National Guard-Health Affairs (MNG-HA), Riyadh, Saudi Arabia; Genetics & Precision Medicine Department, King Abdulaziz Medical City, Ministry of National Guard-Health Affairs (MNG-HA), Riyadh, Saudi Arabia; King Saud bin Abdulaziz University for Health Sciences, King Abdullah International Medical Research Centre, Ministry of National Guard-Health Affairs (MNG-HA), Riyadh, Saudi Arabia; KCBRC, Biological and Environmental Science and Engineering Division (BESE), KAUST, Thuwal, Saudi Arabia; Department of Pathology and Laboratory Medicine, King Abdulaziz Medical City (KAMC), Riyadh, Saudi Arabia; King Saud bin Abdulaziz University for Health Sciences, King Abdullah International Medical Research Centre, Ministry of National Guard-Health Affairs (MNG-HA), Riyadh, Saudi Arabia; Department of Pediatrics, College of Medicine, Qassim University, Qassim, Saudi Arabia; Computational Bioscience Research Center (CBRC), Computer, Electrical and Mathematical Sciences & Engineering Division (CEMSE), King Abdullah University of Science and Technology (KAUST), Thuwal 23955-6900, Saudi Arabia

## Abstract

**Motivation:**

Structural genomic variants account for much of human variability and are involved in several diseases. Structural variants are complex and may affect coding regions of multiple genes, or affect the functions of genomic regions in different ways from single nucleotide variants. Interpreting the phenotypic consequences of structural variants relies on information about gene functions, haploinsufficiency or triplosensitivity and other genomic features. Phenotype-based methods to identifying variants that are involved in genetic diseases combine molecular features with prior knowledge about the phenotypic consequences of altering gene functions. While phenotype-based methods have been applied successfully to single nucleotide variants as well as short insertions and deletions, the complexity of structural variants makes it more challenging to link them to phenotypes. Furthermore, structural variants can affect a large number of coding regions, and phenotype information may not be available for all of them.

**Results:**

We developed DeepSVP, a computational method to prioritize structural variants involved in genetic diseases by combining genomic and gene functions information. We incorporate phenotypes linked to genes, functions of gene products, gene expression in individual cell types and anatomical sites of expression, and systematically relate them to their phenotypic consequences through ontologies and machine learning. DeepSVP significantly improves the success rate of finding causative variants in several benchmarks and can identify novel pathogenic structural variants in consanguineous families.

**Availability and implementation:**

https://github.com/bio-ontology-research-group/DeepSVP.

**Supplementary information:**

Supplementary data are available at *Bioinformatics* online.

## 1 Introduction

Structural genomic variants are genomic variants that affect >50 base pairs and include copy number variants, insertions and deletions (Eichler, 2019). Many structural variants (SVs) are implicated in heritable diseases (Sudmant *et al.*, 2015). While there have been several efforts to predict and prioritize pathogenic genomic variants (Eilbeck *et al.*, 2017), predicting the functional impact of SVs discovered through genome sequencing studies remains challenging due to the diversity of variant size and type; SVs may cover multiple coding and non-coding regions, overlap several genes, and are affected by haploinsufficiency and triplosensitivity (Kidd *et al.*, 2008).

Methods for predicting the pathogenicity of genomic variants may be based on their impact on protein structure, measures of sequence conservation or function (Eilbeck *et al.*, 2017). However, due to the complexity of the SVs, including the variant size, type and overlap with multiple genes, designing methods that determine SV pathogenicity is more challenging. Several efforts for analyzing the clinical impact of SVs have focused on well-matched cases and controls. For instance, by evaluating the loci and the respective pathways that may be impacted by an SV at these loci, it became possible to define novel genes involved in complex disorders such as autism (Pinto *et al.*, 2010) or immune-related disorders (Rossin *et al.*, 2011). While there are several methods to identify disease-associated variants in cohorts, it is more challenging to discover disease-associated variants that exist in a single sample or pedigree, in particular in rare Mendelian disorders (Sanchis-Juan *et al.*, 2018).

Methods that evaluate the functional consequence of SVs in individual genomes use different strategies. Several approaches include genomic information, such as variant length, haploinsufficiency measures or GC contents, to separate pathogenic from benign SVs (Hehir-Kwa *et al.*, 2010; Kleinert and Kircher, 2021; Sharo *et al.*, 2020; Zhang *et al.*, 2021). Furthermore, the predicted pathogenicity of deleterious single nucleotide variants within an SV can be used to estimate pathogenicity of SVs (Ganel *et al.*, 2017). Additionally, phenotypes associated with a loss of function in single genes has also been used for prioritizing SVs (Doelken *et al.*, 2013; Köhler *et al.*, 2014).

Phenotype-driven variant prioritization methods aim to link variants to the phenotypes observed in individuals using prior knowledge (Eilbeck *et al.*, 2017). Commonly, the link is established using a similarity measure between phenotypes associated with a variant or gene and the phenotypes observed in a patient (Smedley *et al.*, 2015). Phenotype-based methods are successful in finding disease-associated variants (Shefchek *et al.*, 2020) but suffer from the limited information about variant– or gene–phenotype associations. One way to overcome this limitation is to utilize phenotypes observed in model organisms and link them to human phenotypes (Shefchek *et al.*, 2020; Smedley *et al.*, 2013); however, even when including phenotypes from model organisms a large portion of human protein-coding genes remain without associations, thereby limiting the success of phenotype-based methods to variants or genes that have previously been studied either in humans or animal models, or relying on guilt-by-associations approaches in which information about phenotypes is propagated through associations such as interaction networks (Smedley *et al.*, 2014).

Several deep learning methods are now available that can predict phenotypes (Kulmanov and Hoehndorf, 2020; Zhou *et al.*, 2019) or associate phenotypes with different types of information available for genes, including functions of gene products and site of expression (Chen *et al.*, 2020; Smaili *et al.*, 2019). These methods use machine learning to relate information through background knowledge contained in ontologies, and can accurately identify phenotype-associated genes without prior knowledge about phenotypes, often significantly improving over the use of semantic similarity measures (Kulmanov *et al.*, 2020). A limitation of these methods is that they are often transductive instead of inductive (Kulmanov *et al.*, 2020), i.e. the diseases or disorders for which associated genes are predicted should already be available at the time of training the model. As these methods require information about disease-associated phenotypes during training, they will therefore not generalize to entirely new cases, thereby limiting their application in identifying phenotype-associated genomic variants.

We developed a machine learning method that predicts whether a copy number variant (i.e. an SV that is either a duplication or deletion) is pathogenic and involved in the development of specific phenotypes. Our method combines genomic information and clinical phenotypes, and leverages a large amount of background knowledge from human and animal models. For this purpose, we extend an ontology-based deep learning method that has previously been applied to identifying gene–disease associations (Chen *et al.*, 2020) to allow it to be applied to patient-specific phenotypes. We demonstrate that our method can improve over the state-of-the-art methods in detecting pathogenic deletions or duplications where phenotypes follow a Mendelian inheritance pattern; in particular, DeepSVP can improve the precision in finding phenotype-associated variants. We further apply our method to the diagnosis of a family with congenital disease involving infantile spasms and seizures for which previous analysis of single nucleotide variants in whole exome and whole-genome sequencing data found no associated variant. We make DeepSVP freely available as a Python package at https://github.com/bio-ontology-research-group/DeepSVP.

## 2 Materials and methods

### 2.1 Inputs, outputs and problem statement

DeepSVP is a machine learning model that takes as input a set of SVs in Variant Call Format (VCF) format together with a set of phenotypes encoded using the Human Phenotype Ontology (HPO). DeepSVP outputs a list of the variants from the input VCF file ranked by their probability of being associated with (or causative of) the set of phenotypes provided as input.

### 2.2 Data sources and ontologies

We use as training and testing dataset the set of pathogenic and benign SVs aligned to the human reference genome GRCh38 obtained from the database of genomic structural variation (dbVar; Griffith and Griffith, 2004) downloaded on February 8, 2020. We use functional and phenotypic characteristics for genes in the human genome, in particular the phenotypes associated with human genes in the HPO database (Köhler *et al.*, 2019), the phenotypes associated with mouse orthologs in the Mouse Genome Informatics database (Bult *et al.*, 2019), the functions of gene products from UniProt (UniProt Consortium, 2019), gene expression in human cell types (Tabula Muris Consortium *et al.*, 2018) and the anatomical site of expression from the GTEx tissue expression database (GTEx Consortium, 2015). These annotations are characterized using the HPO (Köhler *et al.*, 2019), Mammalian Phenotype Ontology (MP; Bult *et al.*, 2019), Gene Ontology (GO; Gene Ontology Consortium, 2019), Cell Ontology (CL; Diehl *et al.*, 2016) and the anatomical site of expression (UBERON) ontology (Mungall *et al.*, 2012). Detailed information about the data sources and ontologies is provided in the Supplementary Section S1.

### 2.3 Training the DeepSVP model

**Fig. 1.**
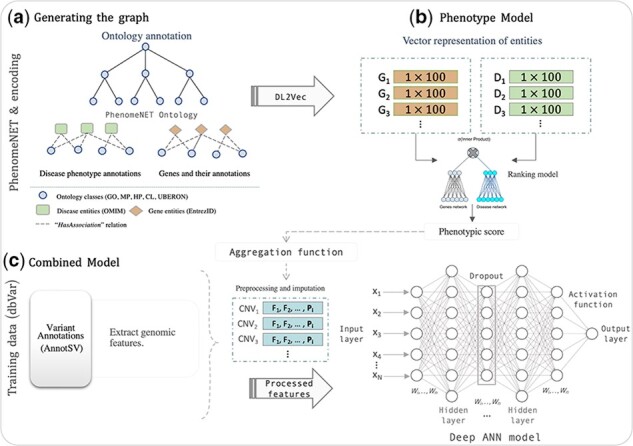
Overview over the DeepSVP model. (**a**) First, a graph is generated from the ontology axioms in which nodes represent classes or entities annotated with ontology classes, and edges represent axioms that hold between these classes. (**b**) The DL2Vec workflow takes a set of phenotypes as input and predicts whether a gene is likely associated with these phenotypes using the background knowledge in the graph generated from the ontology and its associations. (**c**) The combined model uses the prediction score of the DL2Vec phenotype model combined with genomic features derived from SVs. The model outputs a prediction score for each variant that determines how likely the variant is causative of the phenotypes provided as input. G, genes; D, diseases; F, (genomic) features; P, phenotypic score

The DeepSVP model consists of two parts: a phenotype prediction module that matches genes and the phenotypes observed in the affected individuals, and a pathogenicity module which determines whether a variant is disrupting normal gene functions; DeepSVP combines both modules in a joint model. Figure  1 presents a high-level summary of training the DeepSVP model, and a more detailed description of the model and training process is provided in Supplementary Section S2. 

To determine pathogenicity of an SV, we rely on features from public databases, in particular the features provided by AnnotSV version 2.3 (Geoffroy *et al.*, 2018). The variant features generated by AnnotSV either characterize the entire variant (such as the length of the variant or its GC content) or they are derived from genes overlapping the variant (such as the number of genes or length of transcripts). A detailed description of variant-based features is provided in Supplementary Section S3 and Table S2 summarizes all the features used in our predictive model.

The phenotype prediction module uses the DL2Vec (Chen *et al.*, 2020) method to encode ontology-based annotations associated with genes in a low-dimensional feature vector. DL2Vec ‘embeds’ ontologies and their annotations in a real-valued vector space. For this purpose, DL2Vec first generates a graph G=(V,E) from ontology axioms in which nodes represent classes and edges represent axioms that hold between these classes (Chen *et al.*, 2020). Both genes and diseases are then added to *G* as additional nodes and are linked to the ontology classes with which they are annotated. DL2Vec then explores the graph using random walks, and generates embeddings from these walks using Word2Vec. We use HPO to represent the phenotypes associated with diseases, and associate genes with classes from HPO, GO, MP, CL and UBERON. As these annotations are available for a different number of genes, we generate gene embeddings using each ontology separately. As parameters for DL2Vec, we use 100 random walks with a walk length of 25; we use the Word2Vec (Mikolov *et al.*, 2013) skip-gram model to generate the embeddings from the walks with 10 window size, 1 as the minimum count value and an embedding size of 100. We train the skip-gram model for 20 epochs. As a result, we obtain a real-valued feature vector for each disease and gene (and ontology class) of size 100. The embeddings representing genes and diseases are used to train a model that predicts gene–disease associations and outputs a value between 0 and 1 for each gene–disease pair (see Supplementary Section S2). We use this value as a feature in the DeepSVP model. The use of the (HPO-based) disease embedding allows our model to encode the phenotypes that are caused by a pathogenic variant.

For training DeepSVP, we considered as positive instances all the causative variants in our training set with the disease phenotypes for which they are causative. We separate the positive instances from two types of negatives instances: the benign variants which are not implicated in any disease, and pathogenic variants that are not related to the disease phenotypes observed in a patient (but potentially related to another set of phenotypes).

We trained and evaluated our models using a nested cross-validation in which we first divide our data into five folds of which we use one for testing and four for training in each iteration, and then divide the four folds used for training into another two folds of which we use one for training and one for validation and tuning hyperparameters. We stratify all folds by disease, i.e. ensure that variants in each fold are associated with distinct diseases and therefore different phenotypes; this stratification aims to ensure that our model generalizes to new diseases (with new sets of phenotypes) that were not seen during training. We use the training folds for fitting model parameters, the validation fold to tune hyperparameters and report performance on the testing folds.

The architecture of the prediction models is derived by hyperparameter optimization using Scikit-Optimize with Bayesian optimization (skopt) within each training iteration. We provide a detailed description of the set of hyperparameters we tuned and use in Supplementary Section S2.3. We build separate models for each aggregation operation (either maximum or average) and each ontology dataset. For the final DeepSVP model, we chose the hyperparameters that resulted in best testing performance in one of the folds. We find that our model can separate positive from negative cases with an Area Under the Receiver Operating Characteristics Curve (ROCAUC) ranging from 0.8959 using only anatomical site of gene expression as background knowledge to 0.9700 combining all background knowledge; Supplementary Table S3 contains the cross-validation results.

We implemented our models using Keras with a TensorFlow backend, and training was performed on a single Nvidia Tesla V100 GPU. We evaluated the performances of our model in the testing set using the ROCAUC, *F*1-score, the Area Under the Precision–Recall Curve (PRAUC) and the Diagnostic Odds Ratio (DOR; Glas *et al.*, 2003).

#### 2.3.1 Phenotype-based inference in DeepSVP

We use the DeepSVP model to rank SVs depending on their predicted pathogenicity and the relations between genes affected by the SV and the phenotype observed in affected individuals. For inference, DeepSVP takes a VCF file (containing a set of SVs *S*) and a set of phenotypes *P* as input. We use AnnotSV to generate the genomic features for each SV v∈S that the DeepSVP model uses as features. We then generate a new embedding representing the set of phenotypes for which the prediction is made. We generate the embedding by modifying the DL2Vec graph and adding a new node *i*, and adding an edge between *i* and all phenotypes p∈P.

We then generate a new embedding for the new node *i* by performing a sequence of random walks starting at *i* and updating the pretrained skip-gram model used by DL2Vec using these walks. This approach allows the skip-gram model to generate an embedding for a new set of phenotypes while considering the full DL2Vec graph generated from the ontology. Using the trained phenotype prediction model (for each ontology), we then predict the phenotype-based scores used as features by DeepSVP.

### 2.4 Evaluation and comparison of DeepSVP

We evaluate DeepSVP on disease-associated variants added to the dbVar database between February 8, 2020 and July 2, 2020. Our training dataset and that of StrVCTVRE (Sharo *et al.*, 2020) and AnnotSV (Geoffroy *et al.*, 2018) are limited to the set of variants that have been added to dbVar prior to that date; CADD-SV (Kleinert and Kircher, 2021) has been trained with data that may overlap with our test data. In total, 1503 disease-associated variants were added between February 8, 2020 and July 2, 2020, covering 579 distinct diseases; 175 of these diseases are not linked with any variant in our training set. We created synthetic patient samples by inserting a single causative variant into a whole-genome sequence from the 1000 Genomes Project for SV (1000 Genomes Project Consortium, 2012). The set of SVs in 1000 Genomes contains a total of 68 697 variants for 2504 individuals from 26 populations. Using the 1000 Genomes frequencies for all populations, we exclude all variants with Minor Allele Frequency of >1% which results in 2391 variants remaining. Each variant in dbVar is linked to an Online Mendelian Inheritance in Man (OMIM; Amberger *et al.*, 2011) disease; we obtain the phenotypes of the disease from the HPO database (the file phenotype_annotation.tab) and assign these phenotypes to the synthetic genome. We consider the combination of the synthetic genome and HPO phenotypes as a synthetic patient sample. We repeat this for all 1503 causative variants. Evaluation measures consist of determining in how many of the 1503 synthetic patients the correct (inserted) variant was retrieved at rank 1, 10 and 30, as well as the area under the ROC and the precision–recall curves.

We compared the performance of DeepSVP to three related methods that can rank or classify SVs, CADD-SV (Kleinert and Kircher, 2021), StrVCTVRE (Sharo *et al.*, 2020) and AnnotSV version 2.3 (Geoffroy *et al.*, 2018). CADD-SV and StrVCTVRE are SV impact predictors that use a set of genomic features for SVs relating to the conservation, gene importance, coding region, expression and exon structure, trained using a random forest classifier. CADD-SV uses a larger set of variant annotations compared to StrVCTVRE. AnnotSV provides a classification for each SV based on recommendations for the interpretation of copy number variants (Riggs *et al.*, 2020) and classifies variants into pathogenic, likely pathogenic, of uncertain significance, likely benign and benign. AnnotSV can use the phenotype-based method Exomiser (Smedley *et al.*, 2015) to determine whether phenotypes are consistent with previously reported cases, and incorporate the phenotype-based score in the variant classification process. We rank variants based on the class assigned by AnnotSV, descending from pathogenic to benign.

### 2.5 Whole-genome sequencing and SV calling

We collected blood samples for a cohort of individuals which included a Saudi family consisting of five individuals, two unaffected parents, two affected children and one unaffected child (Alfares *et al.*, 2020). We performed whole-genome shotgun sequencing on all individuals. Details on alignment and SV calling are provided in Supplementary Section S4.

### 2.6 Ethical approvals

This study was approved by the Institutional Research Board of the King Abdullah International Medical Research Center (RC16/113 and RC16/211/R2), and the Institutional Bioethics Committee at King Abdullah University of Science and Technology (17IBEC08_Gojobori). All patients have been consented to be enrolled in this study, a written consent form was obtained from all subjects or their parents or legal guardians in the case of minors who are aged 16 years old or younger.

## 3 Results

### 3.1 DeepSVP predicts phenotype-associated SVs

We developed DeepSVP as a method to identify phenotype-associated SVs (deletions and duplications) for patients based on personal genomic data and the phenotypes observed in a patient. The aim of the DeepSVP model is not only to detect potentially pathogenic SVs, but identify the variants that are ‘causative’ for a set of phenotypes observed in a patient. We consider a variant as cause of a set of phenotypes when it is both pathogenic (i.e. disrupts the normal functioning of one or more genes) and contributes to the development of the phenotypes. This approach is motivated by the observation that even healthy individuals may have pathogenic or potentially pathogenic variants that do not result in abnormal phenotypes. Therefore, detecting pathogenicity of a variant alone is typically not sufficient to establish causality (MacArthur *et al.*, 2014).

DeepSVP uses the phenotypes arising from a loss of function in mouse, phenotypes associated with human genes, the anatomical site of gene expression, gene functions and cell types in which genes are expressed, as background knowledge, and links these to the abnormal phenotypes observed in the individual in which the structural was detected. To make predictions based on these different features types, we embed them into a shared representation space using a feature learning method applied to ontologies (Chen *et al.*, 2020). We then combine the resulting embeddings with sequence-derived features that can be used to predict the pathogenicity of a variant, and use a neural network model to predict whether a variant is associated with patient phenotypes. While we evaluated the DeepSVP model using cross-validation (see Section 2.3), an evaluation on a testing dataset that resembles the training data will not be indicative of the performance of the model in a realistic setting where the aim is to identify a disease-associated variant among potentially hundreds or thousands of candidates within a genome.

As a more realistic evaluation of our model, we generate synthetic patient data in which we combine the variants from the genome sequences in the 1000 Genomes Project, insert a single disease-causing pathogenic variant and associate this synthetic genome with the phenotypes of the variant. We then apply our model to all SVs in this synthetic patient, rank the resulting variants based on the DeepSVP prediction score, and evaluate the results (i.e. the rank at which we predict the inserted disease-associated variant). For this evaluation, we select an independent dataset of disease-associated SVs, i.e. a set of variant–disease pairs added to dbVar after we obtained training data for our model. Our evaluation set contains 1503 variants associated with 579 distinct diseases in OMIM and overlapping with 1926 unique genes. There are 175 diseases (associated with 640 variants) that were not present in our training data. We create synthetic patient samples for all variant–phenotype pairs in this evaluation set. We also compare the results of our model with another method for identifying disease-associated variants, StrVCTVRE, CADD-SV and AnnotSV. Table  1 shows the amount of disease-associated variants we identify at different ranks. We evaluate the performance separately for variants that are associated with a disease already present in our training data and variants that were not.

**Table 1.** Summary of the evaluation for predicting causative variants in the benchmark dataset of dbVar, time-based split, for 1503 newly added variants along with the evaluation for 640 newly added variants associated with 175 new diseases which were not present in our training dataset

		Synthetic dataset					Synthetic dataset (novel diseases)				
		Recall@1	Recall@10	Recall@30	ROCAUC	PRAUC	Recall@1	Recall@10	Recall@30	ROCAUC	PRAUC
DeepSVP models using average score	GO	435 (0.2894)	626 (0.4165)	811 (0.5396)	0.9647	0.3303	114 (0.1781)	192 (0.3000)	259 (0.4047)	0.9560	0.2163
	MP	634 (0.4218)	1035 (0.6886)	1217 (0.8097)	0.9850	0.5123	208 (0.3250)	330 (0.5156)	436 (0.6813)	0.9728	0.3873
	HP	545 (0.3626)	977 (0.6500)	1220 (0.8117)	0.9828	0.4528	157 (0.2453)	352 (0.5500)	447 (0.6984)	0.9760	0.3263
	CL	157 (0.1045)	542 (0.3606)	882 (0.5868)	0.9740	0.1761	40 (0.0625)	125 (0.1953)	262 (0.4094)	0.9659	0.1050
	UBERON	254 (0.1690)	602 (0.4005)	1097 (0.7299)	0.9752	0.2377	32 (0.0500)	147 (0.2297)	347 (0.5422)	0.9627	0.1070
	Union	**678 (0.4511)**	1055 (0.7019)	1248 (0.8303)	0.9854	**0.5424**	**221 (0.3453)**	**436 (0.6813)**	**545 (0.8516)**	0.9858	**0.4578**
DeepSVP models using maximum score	GO	325 (0.2162)	536 (0.3566)	725 (0.4824)	0.9558	0.2670	97 (0.1516)	174 (0.2719)	245 (0.3828)	0.9494	0.1917
	MP	237 (0.1577)	630 (0.4192)	855 (0.5689)	0.9605	0.2492	102 (0.1594)	156 (0.2437)	233 (0.3641)	0.9431	0.1949
	HP	445 (0.2961)	**1088 (0.7239)**	**1348 (0.8969)**	**0.9929**	0.4364	122 (0.1906)	370 (0.5781)	528 (0.8250)	**0.9901**	0.3194
	CL	272 (0.1810)	835 (0.5556)	1148 (0.7638)	0.9801	0.2569	52 (0.0813)	250 (0.3906)	390 (0.6094)	0.9756	0.1429
	UBERON	259 (0.1723)	637 (0.4238)	1049 (0.6979)	0.9733	0.2417	69 (0.1078)	161 (0.2516)	369 (0.5766)	0.9656	0.1550
	Union	328 (0.2182)	948 (0.6307)	1122 (0.7465)	0.9750	0.3489	85 (0.1328)	363 (0.5672)	457 (0.7141)	0.9758	0.2585
SV pathogenicity prediction/ranking	StrVCTVRE	72 (0.0479)	223 (0.1484)	405 (0.2695)	0.9178	0.0952	34 (0.0531)	120 (0.1875)	210 (0.3281)	0.9308	0.1142
	CADD-SV	38 (0.0253)	620 (0.4125)	1020 (0.6786)	0.9816	0.1262	9 (0.0141)	162 (0.2531)	373 (0.5828)	0.9871	0.0860
	AnnotSV	19 (0.0126)	229 (0.1524)	700 (0.4657)	0.9605	0.2203	5 (0.0078)	60 (0.0938)	190 (0.2969)	0.9424	0.2319

*Note*: The evaluation inserts one disease-associated SV in a whole genome and reports the rank at which the inserted variant was recovered. Some methods provide the same score for variants, and we break ties randomly and report the absolute number of variants recovered at each rank together with the recall, as well as areas under the ROC curve (using microaverages per genome) and precision–recall curve. Best performing results (using maximum or average score) for each measure are indicated in bold.

The performance using different DeepSVP models varies depending on the availability of the features as well as the gene annotations that are available for each type of feature. The benchmark dataset (i.e. the list of variants we inserted) covers 1920 unique genes, with the largest variant containing 129 genes and the smallest variant containing one gene. There are 635, 963, 1214, 1360 and 493 missing annotations from these genes for features represented using GO, MP, HP, CL and UBERON, respectively. However, the models are still able to predict causative variants using the annotations of the remaining genes (1285, 957, 706, 560 and 1427 for GO, MP, HP, CL and UBERON) which have an annotation and a corresponding representation. The evaluation demonstrates that DeepSVP can identify the variants in novel (i.e. unseen during training) disease-associated variants with one, or more than one, gene. Generally, phenotype-based predictions (using the HP ontology) perform well across most evaluations; predictions based on anatomical site of expression and gene function, for which most data are available and the least number of genes are missing a representation, also perform well across the experiments. We find that DeepSVP using the union with average score significantly improves ranking of disease-associated variants over StrVCTVRE and CADD-SV ($p<6.6\times 10^{-108}$ and $p<1.2\times 10^{-266}$, Mann–Whitney *U* test), methods that use similar features as DeepSVP to determine pathogenicity of variants but do not rely on information about phenotypic or functional consequences. We further evaluated the classifications provided by AnnotSV. AnnotSV classifies variants into five classes (pathogenic, likely pathogenic, unknown significance, likely benign, benign) which we rank by sorting variants based on this classification (from pathogenic variants as highest to benign variants as lowest); and then break the ties randomly. To better compare DeepSVP with AnnotSV’s classification of variants, we perform a reranking experiment in which we apply DeepSVP to all variants in our benchmark set that AnnotSV classifies as either pathogenic or likely pathogenic, i.e. sets of variants that are not further distinguishable using the variant classifications generated by AnnotSV. DeepSVP ranks variants based on their associated phenotypes and pathogenicity, and ranking among pathogenic or likely pathogenic variants demonstrates the improvement provided by DeepSVP over the AnnotSV classifications. Table  2 shows the ROCAUC values for this reranking experiment and demonstrates that DeepSVP’s phenotype-based prioritization can improve over AnnotSV’s ranking.

**Table 2.** Summary of the ROCAUC performance for reranking causative variants from the benchmark dataset with DeepSVP that are assigned the same classification by AnnotSV (930 variants classified as pathogenic, 563 variants as likely pathogenic)

		GO	MP	HP	CL	UBERON	Union
Maximum score	Pathogenic variants	0.9032	0.9032	0.9018	0.9018	0.9034	0.9028
	Likely pathogenic variants	0.9710	0.9711	0.9713	0.9739	0.9704	0.9720
Average score	Pathogenic variants	0.9032	0.9028	0.9029	0.9020	0.9028	0.9016
	Likely pathogenic variants	0.9703	0.9695	0.9704	0.9707	0.9702	0.9694

While we are able to identify disease-associated SVs using phenotype information, the phenotypes reported with a patient-derived sample will not always be complete or as comprehensive as in our evaluation. To determine the effect of different phenotype associations, we further evaluated the performance of DeepSVP when only partial phenotype data is available. We repeat our experiment using synthetic patient samples while randomly removing between 10% and 50% of the phenotypes associated with the sample (Supplementary Tables S4–S7). We find that even when reducing the number of associated phenotypes to 50%, the performance of our model remains comparable; however, when removing phenotype information entirely using the combined model, the predictive performance drops compared to a model that includes information about phenotypes and results in 27 (1.8%) of causative variants ranked first and 575 (38.3%) in the top 10.

### 3.2 DeepSVP identifies a disease-associated variant in a consanguineous family

We applied DeepSVP to investigate whole-genome sequencing data in nine consanguineous families from Saudi Arabia (Alfares *et al.*, 2020) where the clinical presentation is suggestive of genetics underlying etiology with two or more affected individuals, and all previous genetic analyses based on clinical and whole exome analysis were negative. First, we investigated single nucleotide variants and small insertions and deletions as possible explanation of the underlying genetics etiology. However, we were unable to identify a possible disease-associated variant in all nine families using this approach (Alfares *et al.*, 2020).

We applied DeepSVP to rank SVs for possible explanation of the phenotype. In one family, the affected individuals showed hypotonia (HP: 0001290), developmental delay (HP: 0001263), infantile spasms (HP: 0012469), strabismus (HP: 0000486) and seizures (HP: 0001250); after removing all common variations, we ended up with 47 SVs requiring further investigation. DeepSVP ranked one duplication in chromosome 2q24.3(NC¯000002.12: g.164062341¯166264282dup) as top rank using the combined model based on the maximum score (see Supplementary Table S8 for other DeepSVP models); this duplication contains 16 genes (*COBLL1*, *CSRNP3*, *GALNT3*, *GRB14*, *LOC100506124*, *LOC101929633*, *LOC102724058*, *SCN1A*, *SCN1A-AS1*, *SCN2A*, *SCN3A*, *SCN9A*, *SLC38A11*, *SNORA70F*, *TTC21B*, *TTC21B-AS1*). Duplication of 2q24.3, including the cluster of voltage-gated sodium channel genes, is linked with hypotonia, seizures and neonatal epilepsy in several unrelated cases (Firth *et al.*, 2009; Okumura *et al.*, 2011; Simonetti *et al.*, 2012). We further confirmed the variant with length 2 201 941 using Array Comparative Genomic Hybridization in a clinical laboratory. DeepSVP outputs the gene based on which phenotypic similarity was established (using the maximum phenotype score), and among the 16 genes, the *SCN1A* gene has the maximum phenotype score using MP model. The heterozygote loss of function of *SCN1A* in mouse is a model of Dravet syndrome (Kim *et al.*, 2018; Miller *et al.*, 2014) and resembles many of the phenotypes observed in the family we analyzed. Our results shows that DeepSVP can not only identify disease-associated SVs but further yield interpretable results that can provide actionable clinical information. We compared the results with predictions of AnnotSV and StrVCRTRE. AnnotSV classified 11 variants out of 47 as pathogenic, including the variant we identified. StrVCRTRE scored only 6 variants out of 47 (the remaining variants are either <50 bp in length or not exonic, and thereby out of the scope of StrVCRTRE), and the variants we identified ranked 4 out of 6.

## 4 Discussion

### 4.1 Related work

A seminal study investigated the application of phenotype-similarity in CNVs (Doelken *et al.*, 2013) and the results let to the PhenoGramViz method and tool (Köhler *et al.*, 2014). PhenoGramViz, as well as AnnotSV, use phenotypes to rank and prioritize SVs using phenotype information. To overcome the limitation of missing information about phenotypes, PhenoGramViz and AnnotSV rely on mouse phenotypes. While mouse phenotypes increase the coverage of genes with phenotype associations, there are nevertheless a large number of genes for which no phenotype associations are available. DeepSVP overcomes the limitation of missing phenotypes by incorporating information related to genes through ontologies, mainly the functions of gene products, gene expression in individual cell types and anatomical sites of expression and systematically relating them to their phenotypic consequences through ontologies. The phenotype-based prediction model in DeepSVP is modular and can be utilized as part of other methods such as AnnotSV.

PhenoGramViz is not a method that directly prioritizes SVs but relies on visualizing ranking results and exploration by users. While this is useful in targeted studies, DeepSVP can be applied as a component of computational workflows while still enabling interpretation of results. AnnotSV provides a classification rank for each SV using five classes based on their overlap with known variants from different data sources, and aims to implement clinical classification guidelines for variants. DeepSVP, on the other hand, provides pathogenicity prediction for each variant rather than categorize them and includes phenotype prediction models not only to identify relatedness to known phenotypes but also to predict new ones; it may therefore be more suitable for generating hypotheses about phenotype associations of SVs that do not overlap with known disease genes. StrVCRTRE is a method that also directly predicts pathogenicity of SVs and uses similar features related to the gene importance, coding sequence and expression, which allows us to compare directly. A key difference between DeepSVP and StrVCRTRE is DeepSVP’s use of phenotype information while StrVCRTRE does not rely on phenotype information which improves prediction results significantly. Furthermore, StrVCRTRE ranks only the exonic variants, while DeepSVP ranks both exonic and intronic based on the availability of the genomics features. We evaluated and compared DeepSVP with AnnotSV and find that DeepSVP does not improve over AnnotSV with respect to recall but it improves the precision of finding phenotype-associated variants; AnnotSV is not a method to rank variants but rather to classify variants in categories, which may lead to multiple variants being classified as ‘pathogenic’ and therefore decrease precision. DeepSVP ranks variants without ties and shows generally higher precision in our evaluation; it may also allow DeepSVP to more easily be applied in computational workflows where high precision is desirable. Since version 3.0, AnnotSV also provides a more fine-grained ranking of variants from which the variant classification is derived; we did not compare DeepSVP with this version of AnnotSV as it was released after our synthetic, time-based dataset was created. In the future, we intend to provide additional comparisons of DeepSVP with the different outputs of AnnotSV.

### 4.2 Machine learning with semantic background knowledge for variant prioritization

DeepSVP relies on machine learning for predicting pathogenic and phenotype-associated variants. For this purpose, it relies on advances in machine learning with ontologies that incorporate the background knowledge contained in ontologies in the form of axioms and annotations to ontology classes (Kulmanov *et al.*, 2020). Many such approaches convert ontologies into a graph-based form based on syntactic patterns within the ontology axioms and then apply a graph embedding on the resulting graph (Kulmanov *et al.*, 2020). In DeepSVP, we use DL2Vec which includes a large variety of ontology axioms and can significantly improve the phenotype-based prediction of disease genes. While these ontology-based methods rank genes, DeepSVP directly ranks SVs based on the genomic and phenotypic features collected from public databases, and the phenotypes observed in a patient. We precomputed the embeddings for genes based on different features (function, phenotype and expression in cell types and anatomical parts).

Furthermore, we extended the ontology-based machine learning methods to an inductive setting where we can predict associations between genes and individuals that are defined by their phenotypes which are not known at the time of training DeepSVP. We also applied a rank-based normalization, similar to the method applied by PhenoRank (Cornish *et al.*, 2018), and use the resulting score instead of prediction scores of the neural network model; this transformation is useful when predicting relations where one argument remains fixed as it projects prediction scores into the same distribution.

While we implemented a two-step approach in which we first predict associations between genes and patient phenotypes, and second the pathogenicity and phenotypic relatedness of the variant to the patient phenotypes, it may also be possible to design a model that is trained in an end-to-end fashion in the future. The challenge is the potentially open-ended number of genes to consider.

### 4.3 Clinical application and utility

We evaluated the performance of DeepSVP on a series of real genomes from Saudi individuals where the clinical presentation is suggestive of genetic diseases to assess how well we could recover potentially pathological variants in genes already associated with the disease. We used the whole-genome sequencing data from all family members, and we apply family filtering according to the suitable inheritance pattern. We applied DeepSVP to rank SVs for a possible explanation of the phenotype. In one family, our model was able to find the causative variants associated with the patient phenotypes using the combined prediction model that integrates all the phenotypes information, and also to highlight a candidate gene underlying the main phenotypic manifestations. AnnotSV also classified this variant as pathogenic, together with 10 other variants.

We implemented two models for aggregating phenotypic relatedness between genes and patient phenotypes, one using the maximum and another using the average of scores of all genes. These correspond to two different mechanisms through which an SV elicits abnormal phenotypes: the maximum model is applicable when a single gene within the variant is (primarily) causative for the phenotypes, whereas the average model is applicable in the oligogenic case when multiple genes affected by an SV are causative and may contribute different pathologies.

We make DeepSVP freely available to use as a free software command-line tool, including all the steps to train the model. DeepSVP uses as input an annotated VCF file of an individual and clinical phenotypes encoded using HPO. DeepSVP can be used as a part of interpretation workflows in a clinical setting, or incorporated in interactive variant exploration methods.

## Supplementary Material

btaa859_Supplementary_Data

## References

[btab859-B1] 1000 Genomes Project Consortium (2012) An integrated map of genetic variation from 1,092 human genomes. Nature, 491, 56. 23128226 10.1038/nature11632 PMC3498066

[btab859-B2] Alfares A. et al (2020) What is the right sequencing approach? Solo VS extended family analysis in consanguineous populations. BMC Med. Genomics, 13, 103. 32680510 10.1186/s12920-020-00743-8 PMC7368798

[btab859-B3] Amberger J. et al (2011) A new face and new challenges for Online Mendelian Inheritance in Man (OMIM^®^). Hum. Mutat., 32, 564–567. 21472891 10.1002/humu.21466

[btab859-B4] Bult C.J. et al (2019) Mouse Genome Database (MGD) 2019. Nucleic Acids Res., 47, D801–D806. 30407599 10.1093/nar/gky1056 PMC6323923

[btab859-B5] Chen J. et al (2020) Predicting candidate genes from phenotypes, functions and anatomical site of expression. Bioinformatics, 37, 853–860. 10.1093/bioinformatics/btaa879 PMC8248315 33051643

[btab859-B6] Consortium T.M. et al (2018) Single-cell transcriptomics of 20 mouse organs creates a tabula muris. Nature, 562, 367. 30283141 10.1038/s41586-018-0590-4 PMC6642641

[btab859-B7] Cornish A.J. et al (2018) PhenoRank: reducing study bias in gene prioritization through simulation. Bioinformatics, 34, 2087–2095. 29360927 10.1093/bioinformatics/bty028 PMC5949213

[btab859-B8] Diehl A.D. et al (2016) The cell ontology 2016: enhanced content, modularization, and ontology interoperability. J. Biomed. Seman., 7, 1–10. 10.1186/s13326-016-0088-7 PMC4932724 27377652

[btab859-B9] Doelken S.C. et al (2013) Phenotypic overlap in the contribution of individual genes to CNV pathogenicity revealed by cross-species computational analysis of single-gene mutations in humans, mice and zebrafish. Dis. Models Mech., 6, 358–372. 10.1242/dmm.010322 PMC3597018 23104991

[btab859-B10] Eichler E.E. (2019) Genetic variation, comparative genomics, and the diagnosis of disease. N. Engl. J. Med., 381, 64–74. 31269367 10.1056/NEJMra1809315 PMC6681822

[btab859-B11] Eilbeck K. et al (2017) Settling the score: variant prioritization and mendelian disease. Nat. Rev. Genet., 18, 599–612. 28804138 10.1038/nrg.2017.52 PMC5935497

[btab859-B12] Firth H.V. et al (2009) Decipher: database of chromosomal imbalance and phenotype in humans using Ensembl resources. Am. J. Hum. Genet., 84, 524–533. 19344873 10.1016/j.ajhg.2009.03.010 PMC2667985

[btab859-B13] Ganel L. et al (2017) SVScore: an impact prediction tool for structural variation. Bioinformatics, 33, 1083–1085. 28031184 10.1093/bioinformatics/btw789 PMC5408916

[btab859-B14] Gene Ontology Consortium (2019) The gene ontology resource: 20 years and still going strong. Nucleic Acids Res., 47, D330–D338. 30395331 10.1093/nar/gky1055 PMC6323945

[btab859-B15] Geoffroy V. et al (2018) AnnotSV: an integrated tool for structural variations annotation. Bioinformatics, 34, 3572–3574. 29669011 10.1093/bioinformatics/bty304

[btab859-B16] Glas A.S. et al (2003) The diagnostic odds ratio: a single indicator of test performance. J. Clin. Epidemiol., 56, 1129–1135. 14615004 10.1016/s0895-4356(03)00177-x

[btab859-B17] Lappalainen, I. et al. (2013) dbVar and DGVa: public archives for genomic structural variation. Nucleic Acids Res., 41, D936–D941. 10.1093/nar/gks1213., PMC3531204 23193291

[btab859-B18] GTEx Consortium (2015) The genotype-tissue expression (GTEx) pilot analysis: multitissue gene regulation in humans. Science, 348, 648–660. 25954001 10.1126/science.1262110 PMC4547484

[btab859-B19] Hehir-Kwa J.Y. et al (2010) Accurate distinction of pathogenic from benign CNVs in mental retardation. PLoS Comput. Biol., 6, e1000752. 20421931 10.1371/journal.pcbi.1000752 PMC2858682

[btab859-B20] Kidd J.M. et al (2008) Mapping and sequencing of structural variation from eight human genomes. Nature, 453, 56–64. 18451855 10.1038/nature06862 PMC2424287

[btab859-B21] Kim Y. et al (2018) Severe peri-ictal respiratory dysfunction is common in Dravet syndrome. J. Clin. Invest., 128, 1141–1153. 29329111 10.1172/JCI94999 PMC5824857

[btab859-B22] Kleinert P. , Kircher M. (2021) CADD-SV—a framework to score the effects of structural variants in health and disease. *bioRxiv*. https://doi.org/10.1101/2021.07.10.451798. 10.1101/gr.275995.121 PMC8997355 35197310

[btab859-B23] Köhler S. et al (2014) Clinical interpretation of CNVs with cross-species phenotype data. J. Med. Genet., 51, 766–772. 25280750 10.1136/jmedgenet-2014-102633 PMC4501634

[btab859-B24] Köhler S. et al (2019) Expansion of the human phenotype ontology (HPO) knowledge base and resources. Nucleic Acids Res., 47, D1018–D1027. 30476213 10.1093/nar/gky1105 PMC6324074

[btab859-B25] Kulmanov M. , Hoehndorf R. (2020) DeepPheno: predicting single gene loss-of-function phenotypes using an ontology-aware hierarchical classifier. PLoS Comput. Biol., 16, e1008453. 33206638 10.1371/journal.pcbi.1008453 PMC7710064

[btab859-B26] Kulmanov M. et al (2020) Semantic similarity and machine learning with ontologies. Brief. Bioinform., 22, bbaa199. 10.1093/bib/bbaa199 PMC8293838 33049044

[btab859-B27] MacArthur D. et al (2014) Guidelines for investigating causality of sequence variants in human disease. Nature, 508, 469–476. 24759409 10.1038/nature13127 PMC4180223

[btab859-B28] Mikolov T. et al (2013) Efficient estimation of word representations in vector space. *CoRR*, 2013. abs/1301.3781.

[btab859-B29] Miller A.R. et al (2014) Mapping genetic modifiers of survival in a mouse model of Dravet syndrome. Genes Brain Behav., 13, 163–172. 24152123 10.1111/gbb.12099 PMC3930200

[btab859-B30] Mungall C.J. et al (2012) UBERON: an integrative multi-species anatomy ontology. Genome Biol., 13, R5. 22293552 10.1186/gb-2012-13-1-r5 PMC3334586

[btab859-B31] Okumura A. et al (2011) Refractory neonatal epilepsy with a de novo duplication of chromosome 2q24.2q24.3. Epilepsia, 52, e66–e69. 21692795 10.1111/j.1528-1167.2011.03139.x

[btab859-B32] Pinto D. et al (2010) Functional impact of global rare copy number variation in autism spectrum disorders. Nature, 466, 368–372. 20531469 10.1038/nature09146 PMC3021798

[btab859-B33] Riggs E.R. et al (2020) Technical standards for the interpretation and reporting of constitutional copy-number variants: a joint consensus recommendation of the American College of Medical Genetics and Genomics (ACMG) and the Clinical Genome Resource (ClinGen). Genet. Med., 22, 245–257. 31690835 10.1038/s41436-019-0686-8 PMC7313390

[btab859-B34] Rossin E.J. et al (2011) Proteins encoded in genomic regions associated with immune-mediated disease physically interact and suggest underlying biology. PLoS Genet., 7, e1001273. 21249183 10.1371/journal.pgen.1001273 PMC3020935

[btab859-B35] Sanchis-Juan A. et al (2018) Complex structural variants in mendelian disorders: identification and breakpoint resolution using short- and long-read genome sequencing. Genome Med., 10, 95. 30526634 10.1186/s13073-018-0606-6 PMC6286558

[btab859-B36] Sharo A.G. et al (2020) StrVCTVRE: a supervised learning method to predict the pathogenicity of human structural variants. *bioRxiv*. https://doi.org/10.1101/2020.05.15.097048. 10.1016/j.ajhg.2021.12.007 PMC8874149 35032432

[btab859-B37] Shefchek K.A. et al (2020) The Monarch Initiative in 2019: an integrative data and analytic platform connecting phenotypes to genotypes across species. Nucleic Acids Res., 48, D704–D715. 31701156 10.1093/nar/gkz997 PMC7056945

[btab859-B38] Simonetti B.G. et al (2012) Duplication of the sodium channel gene cluster on 2q24 in children with early onset epilepsy. Epilepsia, 53, 2128–2134. 23016767 10.1111/j.1528-1167.2012.03676.x

[btab859-B39] Smaili F.Z. et al (2019) OPA2Vec: combining formal and informal content of biomedical ontologies to improve similarity-based prediction. Bioinformatics, 35, 2133–2140. 30407490 10.1093/bioinformatics/bty933

[btab859-B40] Smedley D. et al (2013) PhenoDigm: analyzing curated annotations to associate animal models with human diseases. Database, 2013, bat025. 23660285 10.1093/database/bat025 PMC3649640

[btab859-B41] Smedley D. et al (2014) Walking the interactome for candidate prioritization in exome sequencing studies of Mendelian diseases. Bioinformatics, 30, 3215–3222. 25078397 10.1093/bioinformatics/btu508 PMC4221119

[btab859-B42] Smedley D. et al (2015) Next-generation diagnostics and disease-gene discovery with the exomiser. Nat. Protoc., 10, 2004–2015. 26562621 10.1038/nprot.2015.124 PMC5467691

[btab859-B43] Sudmant P.H. et al (2015) An integrated map of structural variation in 2,504 human genomes. Nature, 526, 75–81. 26432246 10.1038/nature15394 PMC4617611

[btab859-B44] UniProt Consortium (2019) UniProt: a worldwide hub of protein knowledge. Nucleic Acids Res., 47, D506–D515. 30395287 10.1093/nar/gky1049 PMC6323992

[btab859-B45] Zhang L. et al (2021) X-CNV: genome-wide prediction of the pathogenicity of copy number variations. Genome Med., 13, 1–15. 34407882 10.1186/s13073-021-00945-4 PMC8375180

[btab859-B46] Zhou N. et al (2019) The CAFA challenge reports improved protein function prediction and new functional annotations for hundreds of genes through experimental screens. Genome Biol., 20, 1–23. 31744546 10.1186/s13059-019-1835-8 PMC6864930

